# Images in Spine: A Rare Abnormal Bony Fusion

**DOI:** 10.7759/cureus.13719

**Published:** 2021-03-05

**Authors:** Uma V Mahajan, Kyle B Labak, Collin M Labak, Eric Z Herring, Alia M Hdeib

**Affiliations:** 1 Neurological Surgery, University Hospitals Cleveland Medical Center, Cleveland, USA; 2 Neurological Surgery, University Hospitals Cleveland Medical Center, Case Western Reserve University, Cleveland, USA

**Keywords:** cervical spine, congenital spine disease, cervical vertebral fusion syndrome, kyphotic deformity, klippel-feil syndrome, cervical myelopathy, wasp-waist sign

## Abstract

Klippel-Feil syndrome (KFS) is characterized by failed segmentation of the cervical spine leading to inappropriately fused vertebral bodies. A 64-year-old male with a previous L5-S1 decompression presented with significant neck pain with radiation into the entire right upper extremity and hand. Imaging demonstrated fusion of the vertebral bodies at C2-3, C4-6, and C7-T1 with associated disc bulges at C3-4 and C6-7. Common presentation of KFS includes significant spondylosis and cervical myeloradiculopathy in addition to the classic triad of short neck, low posterior hairline, and restricted neck motion. We present exemplary images of this rare condition to aid clinicians in future diagnoses.

## Introduction

Klippel-Feil syndrome (KFS), also known as cervical vertebral fusion syndrome, is classically known as the triad of a short neck, low posterior hairline, and severe restriction of cervical motion [1]. It is formally defined as congenital fusion of two or more cervical vertebrae, and patients often present to the emergency department due to spontaneous or progressive neurological sequelae caused by the unstable skeletal structure [2]. Although KFS is congenital and occurs during embryonic development, aging can aggravate vertebral bone degeneration, and worsen spinal canal stenosis and cervical spondylopathy [3]. Here, we report on a patient presenting to the spine clinic with a primary concern of neck pain. We present exemplary images of this rare condition to aid clinicians in future diagnoses.

## Case presentation

**Figure 1:**
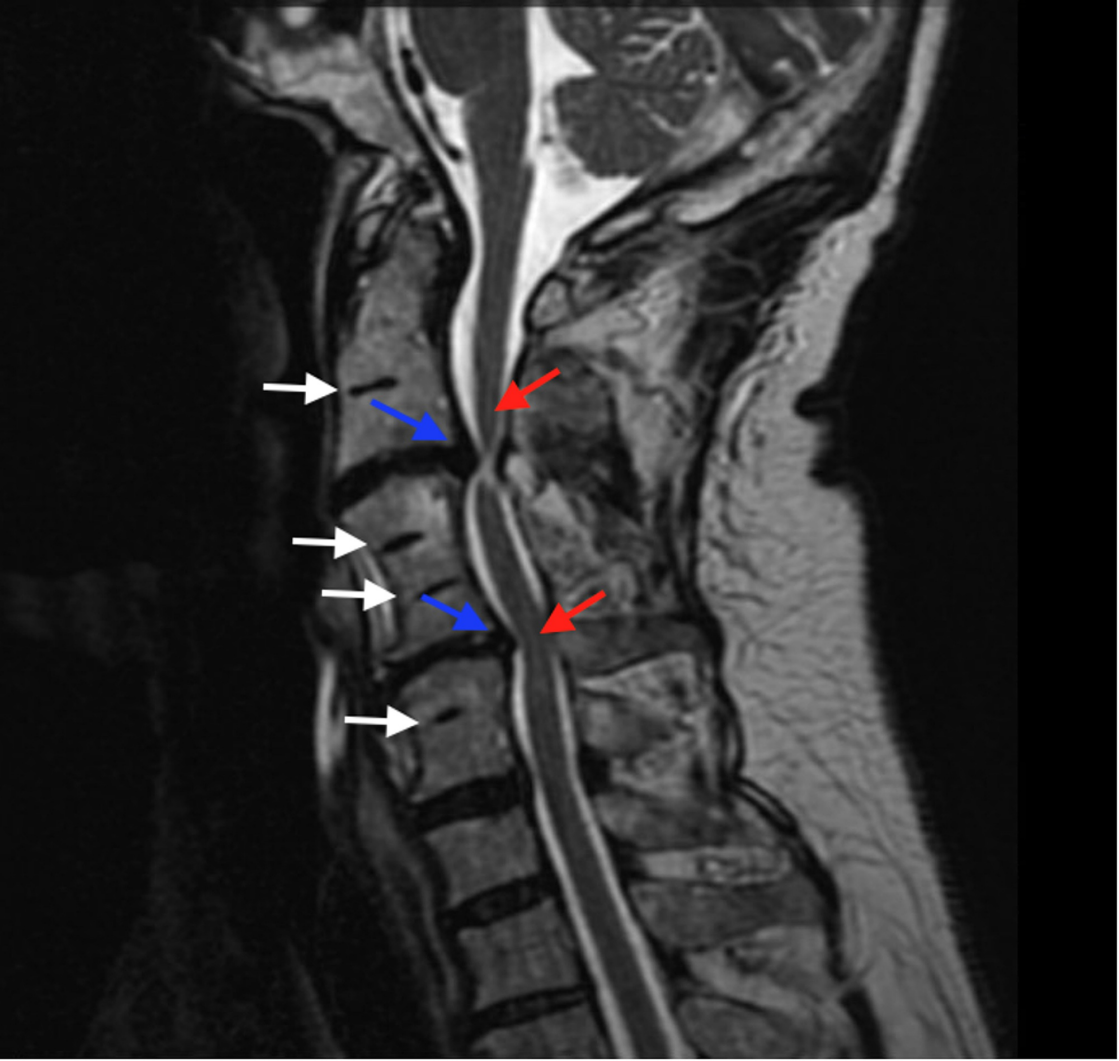
MRI of Cervical Spine. A mid-sagittal MRI T2 sequence of the cervical spine demonstrating multiple vertebral bodies that have auto-fused (white arrows.) Lying between these fused segments are multiple levels of disc bulges (blue arrows) causing significant cord signal change indicative of edema or myelomalacia (red arrows).

**Figure 2:**
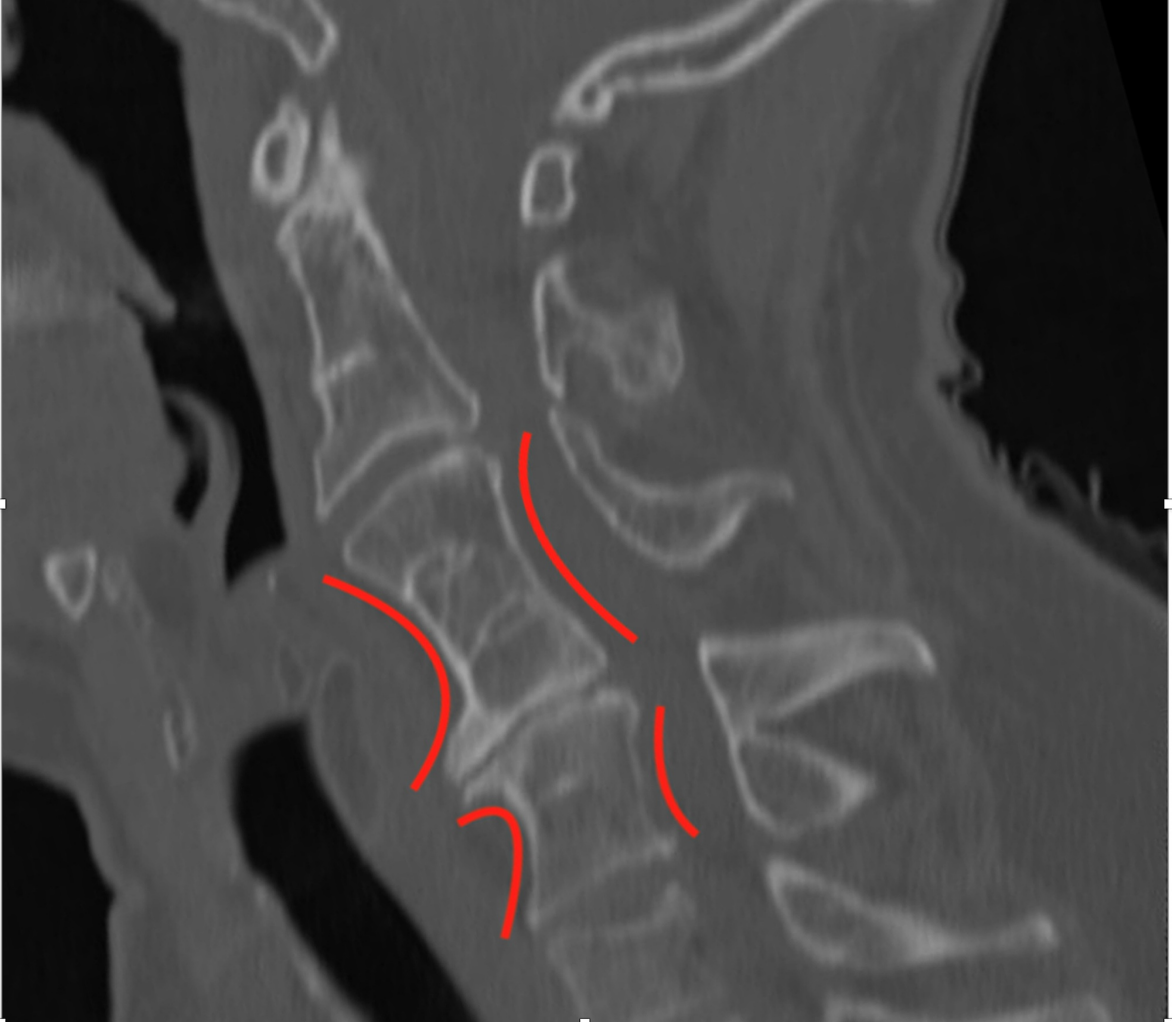
CT of Cervical Spine. A mid-sagittal reformatted CT scan of the cervical spine again showing fusion of multiple vertebral bodies, with associated kyphotic deformity of the cervical spine. Characteristic narrowing of the vertebral bodies, known as the Wasp-Waist sign (red curves) is also seen here.

**Figure 3:**
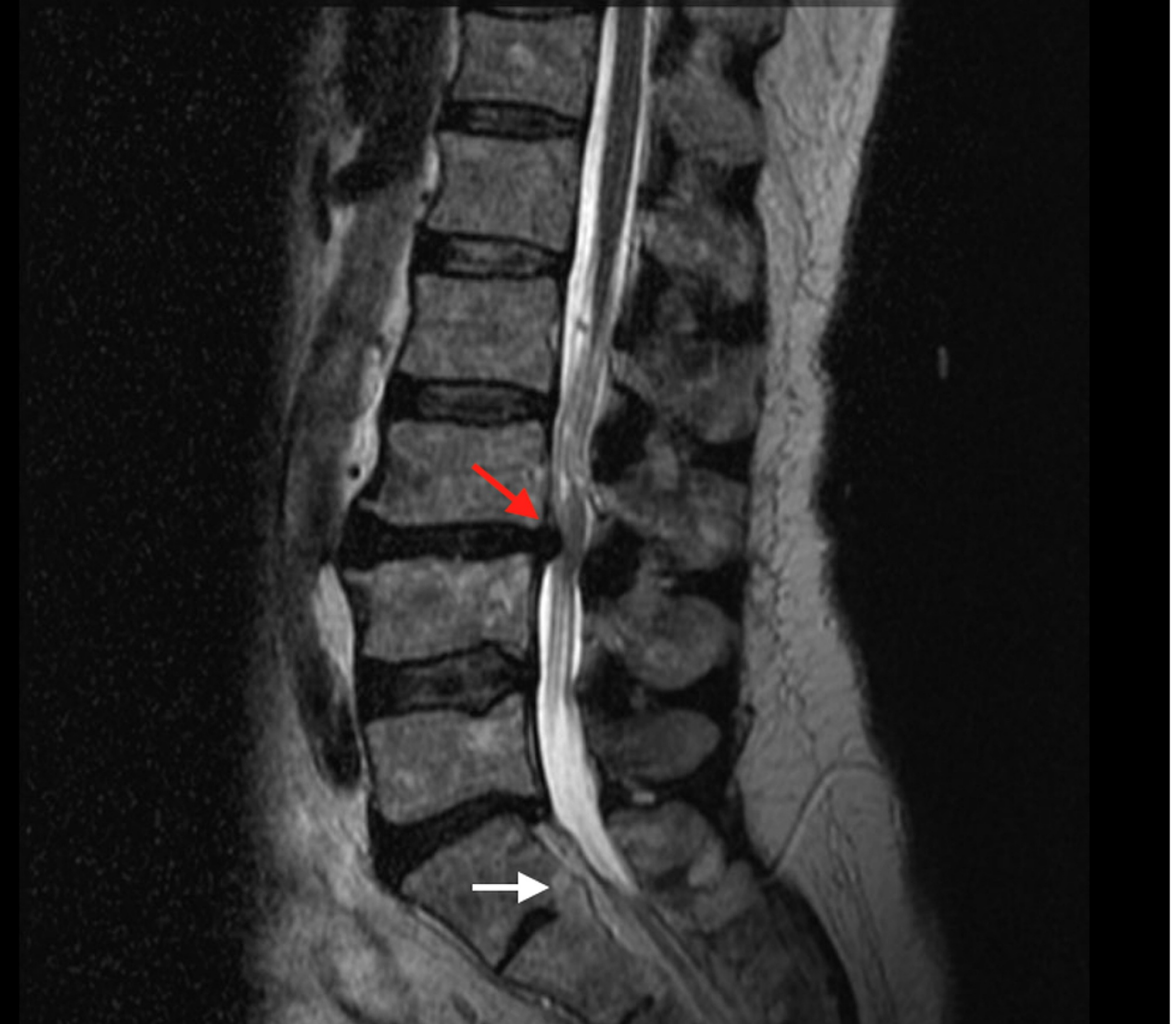
MRI of Lumbar Spine. A mid-sagittal T2-weighted MRI of the lumbar spine with degenerative changes including a disc protrusion at L3-4 (red arrow) and partial auto-fusion of L5-S1 (white arrow), though these may represent surgical changes.

A 64-year-old male presented to our clinic with significant neck pain with radiation into the entire right upper extremity and hand without clear radicular pattern. His past medical history was significant for obesity, a previous L5-S1 decompression at an outside hospital, and alcohol abuse in remission. His pain had progressed and he felt that his arms were beginning to become weaker, causing difficulty using a computer mouse. He also had numbness and tingling, particularly in the right arm and hand. Magnetic resonance imaging (MRI) of the cervical spine was performed, which showed fusion of the vertebral bodies at C2-3, C4-6, and C7-T1 with associated disc bulges at C3-4 and C6-7 causing significant central canal stenosis and spinal cord signal change (Figure 1). Computed tomography (CT) scan of the neck was also performed, which again confirmed this bony fusion at the above cervical levels (Figure 2). MRI of the lumbar spine showed degenerative changes, but was within normal limits (Figure 3). The patient underwent posterior cervical decompression and fusion, with improvement of symptoms and pain.

## Discussion

KFS is characterized by the abnormal fusion of the cervical spine. KFS Type 1 has a single congenitally fused cervical segment, KFS Type 2 has multiple noncontiguous fused cervical segments, and KFS Type 3 has multiple contiguous fused cervical segments [4]. KFS was first identified by Maurice Klippel and André Fiel in 1912, on a radiograph [5]. Tracking the prevalence of KFS is problematized by its heterogeneous presentation, as it may remain undiagnosed for long periods of time. Its prevalence has been estimated to be around 0.0058%, with a slightly higher prevalence in females [6].

Neurological findings often include those consistent with myelopathy, including clonus, hyperreflexia, paresthesias, numbness, and positive Babinski reflex, as these patients tend to develop upper neuron symptoms because of external compression due to posteriorly herniated intervertebral disks. KFS patients may be predisposed to cervical spondylotic myelopathy (CSM), as patients with KFS rely more heavily on segments adjacent to the fused vertebrae [7]. A retrospective study demonstrated KFS patients with hypermobility of the upper cervical spine are at risk for neurological sequelae including pain, whereas those with decreased mobility of the lower cervical spine are at risk for degenerative disease [8].

Heterogeneity in clinical presentation exists within KFS. In a report of 50 patients, fewer than 50% have the classic triad of short neck, low posterior hairline, and limited motion of the neck [9]. Over 50% of KFS patients had scoliosis, and one-third had renal abnormalities. The angle of scoliosis is correlated with the type of KFS (Type 1 is associated with 31°, Type 2 is associated with 9°, and Type 3 is associated with 23°) [10]. Furthermore, a retrospective review of 28 KFS patients with scoliosis found an approximate prevalence of 50%, 21.4%, and 28.6% for Types 1, 2, and 3, respectively [11]. This study also found an approximate female:male prevalence of 5:2. A global patient-reported registry revealed over 25% of KFS patients have Sprengel deformity [12]. Other comorbidities include rib abnormalities, hearing impairment, and genitourinary abnormalities [13]. In rare cases, KFS has been reported in association with a spinal neurenteric cyst [14], craniocervical dermoid cyst [15], and bilateral multilevel cervical ribs and omovertebra [16].

Although etiology is still unclear, some studies posit vascular disruption, global fetal insult, primary neural tube complications, or genetic related factors as relevant possibilities [17]. Mutations in the Pax patterning factor genes, vertebral segmentation genes growth differentiation factor (GDF) 6, GDF 3, and mesenchyme homeobox 1 (MEOX1), notch signalling pathway gene Ripply transcriptional repressor 2 (RIPPLY2), as well as genes bromodomain adjacent to zinc finger domain 1B (BAZ1B), FRAS1‐related extracellular matrix 2 (FREM2), suppressor of fused protein (SUFU), Vang-like protein 1 (VANGL1), and lysine (K)-specific methyltransferase 2D (KMT2D) may be associated with KFS [18-20]. However, its rare and heterogeneous presentation complicates genetic analyses [13].

## Conclusions

Here, we report a case of a 64-year-old male with a previous L5-S1 decompression, who presented with significant neck pain with radiation into the entire right upper extremity and hand. Imaging revealed fusion of the vertebral bodies at C2-3, C4-6, and C7-T1 with associated disc herniations at C3-4 and C6-7, consistent with a diagnosis of KFS. This presentation of spondylosis and cervical myeloradiculopathy is a characteristic of KFS, in addition to the classic triad of short neck, low posterior hairline, and restricted neck motion. Our presented images serve as a clear exemplar of this rare condition. Further work should be done on the etiology and comorbidities of this rare condition.
